# High‐n Phase Suppression for Efficient and Stable Blue Perovskite Light‐Emitting Diodes

**DOI:** 10.1002/advs.202306167

**Published:** 2024-07-11

**Authors:** Piaoyang Shen, Shuo Ding, Zhaobing Tang, Lei Qian, Ting Zhang, Peng Xiao, Tao Chen, Hao Chen, Xinyu Zhang, Yong Ren, Dewei Zhao, Chaoyu Xiang

**Affiliations:** ^1^ Laboratory of Advanced Nano‐Optoelectronic Materials and Devices Qianwan Institute of CNITECH Ningbo Zhejiang 315336 China; ^2^ College of Materials Science and Engineering & Engineering Research Center of Alternative Energy Materials & Devices Ministry of Education Sichuan University Chengdu Sichuan 610065 China; ^3^ Department of Mechanical, Materials and Manufacturing Engineering University of Nottingham Ningbo China Ningbo Zhejiang 315100 China; ^4^ Laboratory of Optoelectronic and Information Materials and Devices Ningbo Institute of Materials Technology and Engineering Chinese Academy of Sciences Ningbo Zhejiang 315201 China; ^5^ Zhejiang Provincial Engineering Research Center of Energy Optoelectronic Materials and Devices Ningbo Institute of Materials Technology & Engineering Chinese Academy of Sciences Ningbo Zhejiang 315100 China; ^6^ Nottingham Ningbo China Beacons of Excellence Research and Innovation Institute University of Nottingham Ningbo China Ningbo Zhejiang 315100 China; ^7^ Key Laboratory of Carbonaceous Wastes Processing and Process Intensification Research of Zhejiang Province University of Nottingham Ningbo China Ningbo Zhejiang 315100 China; ^8^ Hefei National Laboratory for Physical Sciences at Microscale CAS Key Laboratory of Materials for Energy Conversion Department of Materials Science and Engineering School of Chemistry and Materials Science University of Science and Technology of China Hefei Anhui 230026 China

**Keywords:** blue‐emissive, external quantum efficiency, perovskites light‐emitting diodes, quasi‐2D perovskite films

## Abstract

Quasi‐2D perovskites light‐emitting diodes (PeLEDs) have achieved significant progress due to their superior optical and electronic properties. However, the blue PeLEDs still exist inefficient energy transfer and electroluminescence performance caused by mixed multidimensional phase distribution. In this work, transition metal salt (zinc bromide, ZnBr_2_) is introduced to modulate phase distributions by suppressing the nucleation of high n phase perovskites, which effectively shortens the energy transfer path for blue emission. Moreover, ZnBr_2_ also facilitates energy level matching and reduces non‐radiative recombination, thus improving electroluminescence (EL) efficiency. Benefiting from these combined improvements, an efficient blue PeLEDs is obtained with a maximum external quantum efficiency (EQE) of 16.2% peaking located at 486 nm. This work provides a promising approach to tune phase distribution of quasi‐2D perovskites and achieving highly efficient blue PeLEDs.

## Graphical abstract

An efficient quasi‐2D blue PeLEDs at 486 nm are realized by employing transition metal salt (ZnBr_2_). The high Br‐to‐Pb ratio exhibits superior phase distribution control, which suppresses the growth of the high‐n phase and shortens the carrier transfer path between different n phases for highly blue PeLEDs. As a result, the ZnBr_2_‐assisted devices achieve a maximum external quantum efficiency (EQE) of 16.2%.

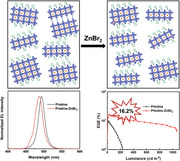

## 1 Introduction

Perovskite light‐emitting diodes (PeLEDs) have recently received significant interest in the next‐generation display technologies, mainly owing to their excellent optical and electronic properties of metal halide perovskite materials, such as tunable emission colors, large carrier mobility, high photoluminescence quantum yields (PLQYs), and high color purity.^[^ ^1^, ^2^, ^3^, ^4^, ^5^, ^6^, ^7^ ^]^ Up to now, metal halide perovskites have enabled major breakthroughs in the external quantum efficiency (EQE) for green‐ and red‐emitting PeLEDs, which have increased to more than 20%.^[^ ^8^, ^9^, ^10^, ^11^, ^12^, ^13^, ^14^ ^]^ Unfortunately, the performance of blue‐emitting LEDs still lags largely behind due to the difficulties in maintaining stable electroluminescence spectra, high quantum efficiency, and operational stability, which undoubtedly limit the application of PeLEDs in full‐color displays and white light illumination.^[^ ^15^, ^16^, ^17^, ^18^, ^19^, ^20^, ^21^ ^]^

Recently, quasi‐2D perovskites have been considered promising candidates to realize efficient and stable blue PeLEDs. Quasi‐2D perovskites with quantum confinement and dielectric confinement effects exhibit higher exciton binding energy and efficient radiative recombination,^[^ ^22^, ^23^, ^24^ ^]^ leading to breakthroughs in quasi‐2D blue PeLEDs.^[^ ^25^, ^26^, ^27^, ^28^ ^]^ It is well known that quasi‐2D perovskites often contain a mixture of phases (especially wide n distribution, n is equal to the number of octahedral layers within a 2D layer), resulting in low emission efficiency because of inefficient internal energy transfer.^[^ ^29^ ^]^ The most commonly used approach to control phase distribution is to introduce more organic cations in quasi‐2D perovskites for the realization of blue emission. Organic materials such as i‐butylammonium bromide (iBABr),^[^ ^30^ ^]^ iso‐propylammonium bromide (IPABr),^[^ ^31^ ^]^ and 1,4‐Bis(aminomethyl)benzene bromide (P‐PDABr_2_)^[^ ^32^ ^]^ can manage the phase distribution to achieve efficient blue emission, however, high amounts of large size organic materials can induce poor carrier transport due to their insulating nature. As compared with the unstable organic additives, the inorganic materials are desired to work as an efficient dimension modulation for further improving the performance of blue PeLEDs. Inorganic materials such as sodium bromide (NaBr) have been used to additionally modulate the distribution of the quasi‐2D perovskites by promoting the growth of large‐n components and passivate A‐site vacancies.^[^ ^33^, ^34^ ^]^ However, the recombination of excitons in high‐n domains will induce undesired redshift of the emission and lack of deep analysis mechanisms of Br^−^. Other inorganic materials such as CsCl have also been introduced to regulate the dimensional crystal structures, but the materials with mixed halogens have poor spectra and device stability.^[^ ^28^ ^]^ Meanwhile, zinc bromide (ZnBr_2_) additives were further applied to mixed halogens to improve spectra stability, but an in‐depth analysis of the role of zinc ion and bromide ion separately in perovskite films are still lacking.^[^ ^35^ ^]^

In this work, we analyze the role of zinc ion and bromide ion separately. The high Br‐to‐Pb (bromide‐to‐lead) ratio shows superior phase distribution control, which disrupts the growth of the high‐n phase. ZnBr_2_‐assisted perovskite films achieve a more concentrated n distribution and shorten carrier transfer path between different n phases for highly blue PeLEDs. We also found that ZnBr_2_ could enhance carrier injection with optimal energy levels and passivate the defect states. Br‐ can effectively alleviate the halide vacancy defects and zinc ion can passivate B‐site vacancies. As a result, high‐performance ZnBr_2_‐assisted quasi‐2D PeLEDs are obtained with a maximum EQE of 16.2% and the emission peak located at 486 nm. Our work provides a promising approach to realizing efficient and stable blue PeLEDs.

## 2 Results and Discussion

**Figure 1:**
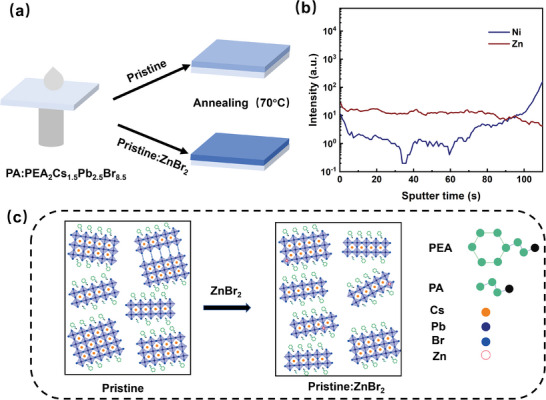
a) Preparation process of quasi‐2D perovskite film with and without ZnBr_2_. b) Time of flight secondary ion mass spectrometry (TOF‐SIMS) profile of Zn^2+^ element distribution (this curve was obtained by eliminating its signal interference). c) Schematic illustration of the n‐value phase distribution of quasi‐2D perovskite after ZnBr_2_ diffusion.

We synthesized blue emission perovskites by mixing different molar ratios of ZnBr_2_, lead bromide (PbBr_2_), cesium bromide (CsBr), phenethylammonium bromide (PEABr) and propylammonium bromide (PABr) in dimethyl sulfoxide (DMSO) and using a one‐step spin‐coating method without antisolvent treatment (see Methods). The preparation process is displayed in **Figure** **1**a. To investigate the role of the Zn cations in the perovskite film, time‐of‐flight secondary‐ion mass spectrometry (TOF‐SIMS) was performed to detect the distribution of the Zn cations. As shown in Figure 1b, Interestingly, the Zn signal is found in the whole pristine:ZnBr_2_ perovskite (denoting ZnBr_2_ is incorporated into the pristine perovskite) film and the signal changes slightly with sputtering etching time, which indicates that Zn cations are uniformly distributed in the perovskite film (It can be clearly seen from Figure S1, Supporting Information). Inevitable wide phase distribution is formed in the pristine perovskites, which results in an inefficient energy transfer process between these quasi‐2D perovskite layers (Figure 1c). For the pristine:ZnBr_2_ perovskite film, the high‐n phase can be suppressed, and the energy level gradient between different phases is shortened through the high Br‐to‐Pb ratio. The phase crystallization process is critical to the final phase distribution of the quasi‐2D perovskite film. A high Br‐to‐Pb ratio can control the phase of 2D perovskite with different thicknesses or different n values, leading to lower n‐phase formation. A detailed logical derivation of how it happens was provided. The pristine perovskite film was fabricated by using the initially prescribed stoichiometric ratio of perovskite components, composed of A cations (inclusive of L‐site), B cations, and X‐site anions. However, upon introducing an excess of X‐anions (bromine), the composition deviated from the intended balance. This surplus of X‐anions disrupts the formation dynamics of the pristine perovskite structure, instigating a competitive process for X sites within the lattice. Specifically, multiple X‐anions cluster around the lead sites, interfering with the establishment of the pristine ABX configuration. This phenomenon impedes further association and hinders the process toward the high n‐phase stage. Here, we introduce ZnBr_2_ to obtain smaller n value and inhibit the formation of high n‐phase. The negative correlation between the perovskite size and the Br‐to‐Pb ratio ([Br−]) has been confirmed by a simplified model,^[^ ^36^ ^]^ as the ratio of Br‐to‐Pb increases, smaller crystal sizes are obtained, which is beneficial for the formation of low‐n phases. This optimized phase distribution could facilitate the formation of cascade energy levels and promote efficient charge transfer/transport in quasi‐2D perovskite layers. In order to conduct an in‐depth analysis of the role of ZnBr_2_, in‐situ absorption measurements during spin‐coating were performed to the perovskite crystallization kinetics and the control of quasi‐2D phase distribution (Figure S5, Supporting Information). The introduction of ZnBr_2_ effectively controls the process of perovskite crystallization kinetics. In Figure S5a,b (Supporting Information), it can be seen that the pristine perovskite film rapidly produces more n = 2 and n = 3 phase, while the pristine:ZnBr_2_ perovskite film grows slowly to form n = 2. In addition, the introduction of ZnBr_2_ also reduces the distribution of high‐n phases (n = 3). In order to observe the proportion of different n phases clearly, we made an intensity change curve of quasi‐2D perovskite formation without and with ZnBr_2_ during spin‐coating (reference baseline: n = 2, Figure S5c,d, Supporting Information). It can be seen that the pristine perovskite film is dominated by n = 2 and n = 3 phase. However, in pristine:ZnBr_2_ perovskite film, the intensity of the n = 3 phase is weak, and the phase is dominated by n = 2.

**Figure 2:**
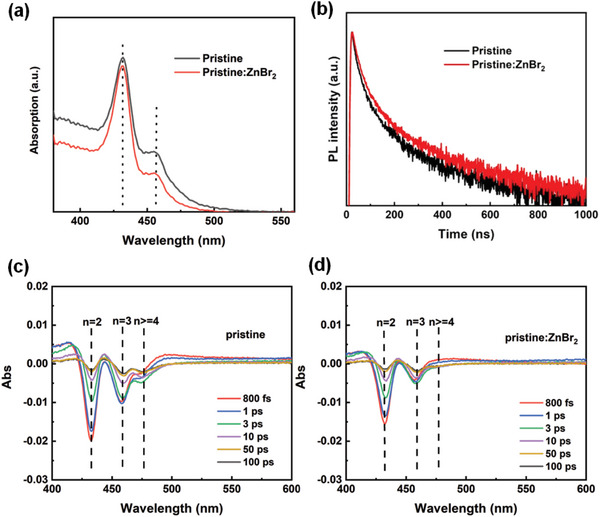
a) Absorption spectra of perovskite films coated on ITO/NiOx/PVK substrate. b) Time‐resolved photoluminescence (TRPL) decay spectra of the pristine and pristine:ZnBr_2_ perovskite films coated on ITO/NiO/PVK substrate. c) Transient absorption (TA) spectra at different probe delay times for pristine perovskite film. d) TA spectra at different probe delay times for pristine:ZnBr_2_ perovskite film.

The UV–vis absorption spectra of the pristine and pristine:ZnBr_2_ perovskite films are shown in **Figure** **2**a. The absorption spectra show the shoulders at ≈433 nm (n = 2) and 452 nm (n = 3) are found for both pristine and pristine:ZnBr_2_ perovskite films. The photoluminescence (PL) spectra of the pristine and pristine:ZnBr_2_ perovskite films are shown in Figure S3 (Supporting Information). Upon the modification of ZnBr_2_, the pristine and pristine:ZnBr_2_ perovskite films have a main PL peak located at ≈492 and 484 nm, respectively, indicating a slight blue‐shift by ≈8 nm for the pristine:ZnBr_2_ perovskite, which probably results from the suppression of high‐n phase by adding high Br‐to‐Pb ratio in quasi‐2D perovskites. The PL quantum yield (PLQY) of the pristine perovskite film increases from 46% to 64% after the introduction of ZnBr_2_ additive, indicating the effective defect passivation (Figure S10, Supporting Information). The absorption spectrum of quasi‐2D perovskite films with different ZnBr_2_ doping amounts (5%, 10%, 15%) was provided in Figure S9 (Supporting Information), When the ZnBr_2_ content gradually increases to 15%, small n phase also increases (n = 1 is the most obvious). Time‐resolved photoluminescence (TRPL) measurements were further performed to investigate the effect of ZnBr_2_ on the carrier lifetime in the perovskites as shown in Figure 2b. The PL decay curves can be fitted by biexponential function models (*I* (*t*) = *I*_0_ + *a*_1_ exp(‐*t*/*τ*_1_) + *a*_2_ exp(‐*t*/*τ*_2_)), where *a*_1_, *a*_2_ are the amplitudes; *τ*_1_, *τ*_2_ represent fast and slow decay lifetimes. The fast time constant is related to the nonradiative recombination of the traps, and the slow time is associated with the radiative recombination.^[^ ^37^, ^38^ ^]^ The fitted parameters for TRPL decay curves are extracted in Table S1 (Supporting Information). It is revealed that both the fast and slow decay lifetimes for pristine:ZnBr_2_ perovskite film of 16.19 and 25.58 ns are much longer than that of pristine perovskite film (*τ*_1_, 13.17 ns, and *τ*_2_, 14.30 ns), which promotes the average fluorescence lifetime of pristine:ZnBr_2_ perovskite (*τ*_ave_ = 41.77 ns) higher than the pristine perovskite (*τ*_ave_ = 27.47 ns). The PL and TRPL results show that the incorporation of ZnBr_2_ can effectively passivate trap states and improve the radiative recombination in the perovskite films.

We further performed transient absorption (TA) spectroscopy measurements to evaluate the effect of ZnBr_2_ on the charge carrier dynamics. The TA spectra of the pristine and pristine:ZnBr_2_ perovskites at different timescales are displayed in Figure 2c,d. In the pristine perovskite films, three distinctive photobleaching (PB) peaks are observed, which are located at 433, 452, and 474 nm, corresponding to n = 2, n = 3, and n ≥ 4 phases consistent with the absorption spectra. On the contrary, in the pristine:ZnBr_2_ perovskite film, two distinctive photobleaching (PB) peaks are observed, which are located at 433 and 452 nm, corresponding to n = 2 and n = 3 phases, i.e., photobleaching (PB) peak at 474 nm (n ≥ 4 phases) disappears. It can be found that the photogenerated carriers are mainly formed in low order (i.e., n = 2, n = 3) phases with the rapid accumulation of n = 2 and n = 3 PB peaks at the initial stage. When decay time is extended, the low‐n PB peaks start to decrease accompanied with a gradual growth of the high‐n PB peak, which indicates the carrier transfer from the larger bandgap phases to the smaller bandgap. Meanwhile, the high‐n (n ≥ 4) PB peak almost disappears for the pristine:ZnBr_2_ perovskite films. Thus, this confirms that a high Br‐to‐Pb ratio can effectively suppress high‐n phase and further shorten the energy transfer path. To determine whether the phase distribution benefits from a high Br‐to‐Pb ratio or Zn^2+^, we considered the effects of the ions on the perovskite film separately. We conducted the steady‐state absorption spectra of Zn^2^^+^‐treated quasi‐2D perovskite film to analyze the changes in phase distribution (Figure S7, Supporting Information). We have conducted comprehensive considerations and utilized representative inorganic and organic zinc salts to validate the effects of zinc ions. Zinc acrylate and Zinc sulfate are chosen to include the Zn^2+^ without Br^−^. As shown in Figure S7 (Supporting Information), the absorption peak position is almost not shifted after adding zinc acrylate and zinc sulfate. The result indicated that Zn^2+^ cannot control phase distribution. Therefore, phase distribution benefits from a high Br‐to‐Pb ratio. To further analyze the role of ZnBr_2_, X‐ray photoelectron spectroscopy (XPS) measurements were performed to confirm the interaction between the ZnBr_2_ and the Pb‐Br framework. The peaks at 143.3 and 138.4 eV for the pristine perovskite are assigned to the Pb 4f signal, which shift toward lower binding energy upon incorporation of ZnBr_2_ and the Br 3d_3/2_ and 3d_5/2_ peaks slightly shifted to lower binding energies, (Figure S6, Supporting Information). The reduced binding energy indicates the lowered oxidation state of lead due to effective surface passivation by ZnBr_2_. which is indicative of less undercoordinated Pb^2+^ due to effective surface passivation by ZnBr_2_. Addressing the issue of perovskite defects is instrumental in fabricating high‐performance perovskite LEDs. Notably, the presence of halide vacancy defects can be effectively mitigated through the use of ZnBr_2_ additives with a high Br ratio. Regarding the passivation of B‐site vacancies, we have established a surface model centered around Pb vacancies and Zn passivated Pb vacancies (Figure S8, Supporting Information). The density functional theory (DFT) calculation method is provided in the experimental section. Utilizing DFT simulations, we calculated the binding energy (E_binding_) of Zn^2+^ to the Pb vacancy site as −5.84 eV. This indicates that Zn^2+^ has an excellent passivation effect on Pb vacancies.

**Figure 3:**
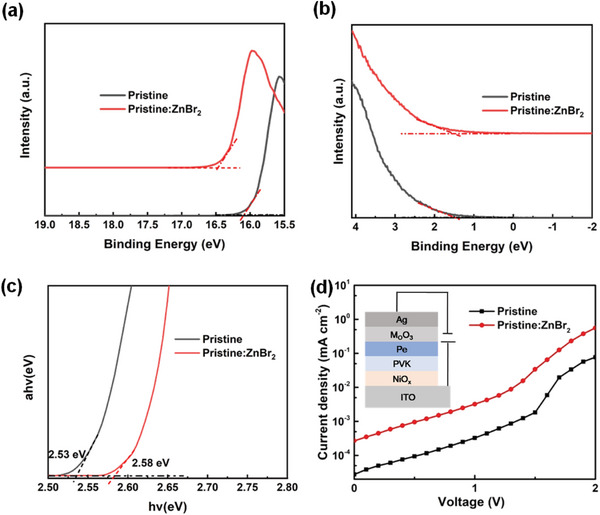
Ultraviolet photoelectron spectroscopy (UPS) results of pristine and pristine:ZnBr_2_ perovskites film, a) high‐binding energy secondary‐electron cutoff edges and b) valence band edge regions. c) Tauc plots show the dependence of (α*hv)^2^* of perovskite films upon the incident photon energy (*hv*) (assuming direct allowed transitions). d) Current density–voltage (*J–V*) curves of the hole‐only device measured under the dark conditions with the structure of ITO/NiOx/PVK/perovskite (pristine, pristine:ZnBr_2_)/MoO_3_/Ag.

To reduce the injection barrier at the charge transport layer (CTL)/perovskite emitting layer, either lowering the VBM of the hole transport layer (HTL) or upshifting the VBM of the perovskite film is highly desirable for the improvement of device performance. The choice of HTL conjugated polymers (e.g., PVK, poly‐TPD, TFB) used for PeLEDs fabrication is quite limited, which is mainly attributed to the strong hydrophobicity of the surface of these charge transport layers. Therefore, upshifting the VBM of the perovskite film is another best choice. For blue PeLEDs with the PVK HTL and the TPBI electron transport layer (ETL), the large band offset at the CTL/perovskite interface limits the device performance due to the deficient carrier injection. In order to investigate the energy band alignment between the perovskite material with or without ZnBr_2_ and the HTL, we performed Ultraviolet photoelectron spectroscopy (UPS) measurement to probe the electronic potentials of perovskite film after incorporating ZnBr_2_. The work functions of the perovskite film were determined from the secondary electron onset region (**Figure** **3**a), where the intercepts of 16.2 and 16.45 eV correspond to the pristine, pristine:ZnBr_2_ films, respectively. In Figure 3b, an E_onset_ of 1.45 eV was nearly independent of the incorporation of ZnBr_2_. Based on the semiconductor band structure, the VBM was 6.45 and 6.2 eV for the samples without ZnBr_2_ and with ZnBr_2_, respectively. Then, the CBM levels of the perovskite films were determined by using the above VBM values and UV–vis absorption spectra (Figure 3c). The pristine:ZnBr_2_ film shows a significant upshift of the VBM by 0.25 eV as compared to that of pristine film, which effectively facilitates a more efficient hole injection from HTL to EML due to the reduced hole injection barrier. The reduction of the perovskite VBM is presumably correlated to the stronger metal activity of Zn than Pb. To better understand the role of ZnBr_2_, the hole‐only devices for the pristine and pristine:ZnBr_2_ perovskite were fabricated to analyze quantitatively the carrier injection (Figure 3d). The result shows that the current density measured for the pristine‐based hole‐only device is approximately two orders of magnitude lower than that of the pristine:ZnBr_2_ one. The increase in current density indicates efficient hole injection after Zn^2+^ incorporation, which is in good agreement with the UPS results.

**Figure 4:**
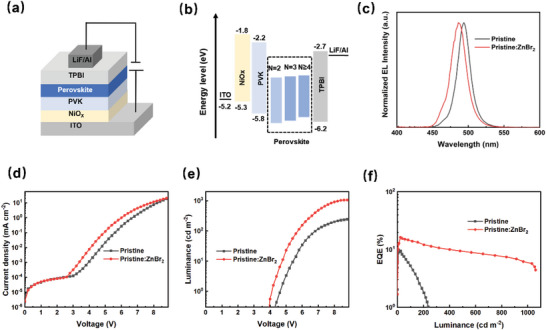
a) Device structure of the PeLEDs: ITO/NiOx/PVK/perovskite/TPBI/LiF/Al. b) Energy band diagram of the PeLEDs; The schematic diagram of perovskites with different n‐phases comes from references,^[^ ^39^, ^40^ ^]^ the energy levels for NiOx, PVK, and TPBI are taken from references.^[^ ^34^ ^]^ c) Normalized EL spectra of the pristine and pristine:ZnBr_2_ PeLEDs at an applied voltage of 5.5 V. d) *J–V* curves, e) *L–V* curves, and f) *EQE–L* curves of the pristine and pristine:ZnBr_2_ PeLEDs.

We fabricated the PeLEDs with a structure of ITO/NiOx/PVK/perovskite/TPBi/LiF/Al, where the perovskite is the emissive layer, NiOx, PVK, and TPBi are the hole injection layer (HIL), hole transport layer (HTL), and electron transport layer (ETL), respectively. ITO and LiF/Al act as anode and cathode, respectively (**Figure** **4**a). The energy level diagram of the whole device is shown in Figure 4b. The EL spectra of the pristine and pristine:ZnBr_2_ PeLEDs exhibit an emission peak at 494  and 486 nm regardless of bias voltage, respectively (Figure 4c). Scanning electron microscope (SEM) images of the perovskite films on the top of ITO/NiOx/PVK are displayed in Figure S2 (Supporting Information). After incorporating ZnBr_2_, the formed perovskite film shows significant improvement of the surface coverage, and few pinholes are observed. The smooth and dense perovskite film should benefit the suppression of current leakage, while the current density–voltage (*J–V*) curves of blue PeLEDs with and without ZnBr_2_ are presented in Figure 4d. The current injection and electroluminescence performance have been improved after the incorporation of ZnBr_2_. The PeLEDs with ZnBr_2_ passivation exhibit stronger luminance than that of the pristine one in the whole voltage range and reach the maximum luminance of 1060.7 cd m^−2^, much higher than 375.2 cd m^−2^ for the pristine PeLEDs (Figure 4c). Importantly, there is a dramatic increase in EQE from 9.1% of the pristine PeLEDs to 16.2% of the pristine:ZnBr_2_ PeLEDs (Figure 4f), which is one of the highest EQE values of blue‐emitting PeLEDs (Table S2, Supporting Information). We attribute the improved device performance to enhanced carrier balance in the modified perovskite film.

Operational stability is also a challenge for blue PeLEDs. We conducted a time‐dependent EL trace under a constant driven current of 0.1 mA cm^−2^ (current density at peak EQE). We recorded the corresponding evolution of luminance, the half‐lifetime (T_50_) defined as time when the luminance declines to 50% of the initial value, in which T_50_ was measured to be 600 s, eight times higher than that of the control device (Figure S4, Supporting Information). The increased operating stability benefits from the Zn^2+^‐induced carrier balance, while Zn^2+^ ions have little effects on the stability.

## 3 Conclusion

We have demonstrated a facile strategy of restricting high‐n phase nucleation and stabilizing low‐n phase to achieve efficient blue PeLEDs. By simply adding ZnBr_2_, the distribution of phase in quasi‐2D blue perovskite film can be modulated with improved charge transport ability and shortened energy transfer path. The resulting PeLEDs show a high peak EQE of 16.2% at 486 nm and excellent spectral stability. Our work provides a useful approach to realizing high‐performance blue PeLEDs.

## 4 Experimental Section

### Materials

Nickel acetate tetrahydrate(99%) was purchased from Acros, ethanolamine (99%) were from Aladdin, ethanol (water ≤ 50 ppm) was purchased from MERYER. Dimethyl sulfoxide and chlorobenzene(CB, 99.8%) were from Macklin, PABr, PEABr, TPBI, and LiF were purchased from Xi'an Polymer Light Technology. Cesium trifluoroacetate, poly(vinylcarbazole), PbBr_2_ were purchased from Sigma–Aldrich, respectively. All materials were used as received.

### Preparation of Precursor Solution

Nickel acetate tetrahydrate (24.4 mg) and 6.1 µL of ethanolamine were dissolved into 1 mL of ethanol, which was stirred at 70 °C for 2 h to form a stable Nickel oxide sol–gel solution. Poly(vinylcarbazole) were dissolved into chlorobenzene with the concentrations of 14 mg mL^−1^. The perovskite precursor solution was prepared by adding PABr, PEABr, CsBr, and PbBr_2_ were dissolved in anhydrous DMSO at a molar ratio of 1.5:4:3:5, with concentrations of 0.15 mmol mL^−1^ (the concentration of Pb^2+^). The ZnBr_2_‐based precursor solution was prepared by mixing with different ratios (5 mmol%/10 mmol%/15 mmol%, molar ratio = ZnBr_2_/PbBr_2_) to obtain precursor solutions. Mixtures were stirred overnight and filtered through polytetrafluoroethylene filters (0.45 µm pore size) before using.

### PeLED Fabrication

Patterned ITO substrates were cleaned with deionized water, acetone, and isopropanol for 30 min, respectively. After drying under nitrogen flow, the substrates were treated with UV ozone for 15 min. The nickel oxide precursor solution was spin coated onto the ITO substrates at a speed of 4000 rpm for 40s, and then annealed at 300 °C for 1 h in air; Then, after cooling and UV 5 min, the samples were transferred into a vacuum chamber, PVK (14 mg mL^−1^) was spin coated at 3000 rpm for 30s, and then annealed at 150 °C for 20 min. Perovskite precursor solution was spin coated at 4000 rpm for 40s, and then baked at 70 °C for 10 min. Then TPBI (40 nm), LiF (1 nm), and Al (100 nm) layers were sequentially deposited by thermal evaporation under a pressure <4 × 10^−4^ Pa.

### Characterization

The current density–voltage–Luminance (*J–V–L*) and EQE results of PeLEDs were obtained by a system with a Keithley 2400 SourceMeter unit and a calibrated commercial LED performance analysis system. The VBM of the perovskite layer was assessed by UPS spectra (ESCALAB 250Xi, ThermoFisher Scientific Inc., USA, He I light source and a hemispherical analyzer). The spectra were referenced to and aligned at the Fermi level of the system determined by measurements on an Au reference. The VBM could be expressed as VBM = 21.22–(E_cutoff_−E_∆_) (where E_∆_ is the gap between the VBM level and the Fermi level (E_F_)).

### DFT Simulation

Ab initio simulation was performed with Cambridge Sequential Total Energy Package (CASTEP, version 20.1.1).^[^ ^41^ ^]^ All the geometry optimization processes were conducted with generalized gradient approximation (GGA) Perdew–Burke–Ernzerhof (PBE) exchange‐correlation functional^[^ ^42^ ^]^ with Koelling–Harmon relativistic treatment, and on the fly generated (OTFG) ultrasoft pseudopotential was applied for better accuracy and consistency. Also, the plane wave basis cut‐off energy was set at 350 eV, and the self‐consistent convergence total energy was chosen to be as 5 × 10^−7^ eV atom^−1^ where maximum force, maximum displacement and maximum stress were set to be as 0.05 eV Å^−1^, 1 × 10^−3^ Å and 0.02 GPa respectively unless specified. 4 × 4 × 4 Monkhorst‐Pack k‐point grids were applied for perovskite lattice and molecules geometry optimization, while 2 × 2 × 4 superlattice surface models with 20 Å vacuum slab were further geometrically optimized by no less than 2 × 2 × 1 k‐point sampling. The binding energy was defined by the formula of *E_binding_* =  |*E*_*Perovskites* *with* *ion*_ − (*E_ion_* + *E_Perovskite_*)|.

## Conflict of Interest

The authors declare no conflict of interest.

## Author Contributions

P.S. and S.D. contributed equally to this work. C.X. and P.S. conceived the study. P.S. fabricated and characterized the PeLEDs devices. P.S., S.D., and Z.T. analyzed and discussed the characteristics of film and device. X.P. and C.T. help P.S. carried out Transient absorption spectra test. H.C. and X.Z. help project design. Y.R., provide funding support. C.X., D.Z., and L.Q supervised this project. All authors discussed the results and commented on the paper.

## Supporting information

Supporting Information

## Data Availability

The data that support the findings of this study are available in the supplementary material of this article.
