# Supplementary material for: High‐n Phase Suppression for Efficient and Stable Blue Perovskite Light‐Emitting Diodes
Source: Adv Sci (Weinh). 2024 Jul 11;11(34):2306167. doi: 10.1002/advs.202306167 (PMC11425891; doi:10.1002/advs.202306167)
Supplement: Supplementary file 1 — Supporting Information [file ADVS-11-2306167-s001.docx]

***Supplementary Information***

**High-n Phase Suppression for Efficient and Stable Blue Perovskite Light-Emitting Diodes**

Piaoyang Shen^1,2,4,9^, Shuo Ding^1,3,4,9^, Zhaobing Tang^1,4,5^, Lei Qian^1,4,5^, Ting Zhang^1,4,5^, Peng Xiao^8^, Tao Chen^8^, Hao Chen^3^, Xinyu Zhang^3^, Yong Ren^3,6,7^*, Dewei Zhao^2^*, Chaoyu Xiang^1,4,5^*

1. Laboratory of Advanced Nano-Optoelectronic Materials and Devices, Qianwan Institute of CNITECH, Ningbo, Zhejiang, 315336, China

2. College of Materials Science and Engineering & Engineering Research Center of Alternative Energy Materials & Devices, Ministry of Education, Sichuan University, Chengdu, Sichuan, 610065, China

3. Department of Mechanical, Materials and Manufacturing Engineering, University of Nottingham Ningbo China, Ningbo, Zhejiang, 315100, China

4. Laboratory of Optoelectronic and Information Materials and Devices, Ningbo Institute of Materials Technology and Engineering, Chinese Academy of Sciences, Ningbo, Zhejiang, 315201, China

5. Zhejiang Provincial Engineering Research Center of Energy Optoelectronic Materials and Devices, Ningbo Institute of Materials Technology & Engineering, Chinese Academy of Sciences, Ningbo, Zhejiang, 315100, China

6. Nottingham Ningbo China Beacons of Excellence Research and Innovation Institute, University of Nottingham Ningbo China, Ningbo, Zhejiang, 315100, China

7. Key Laboratory of Carbonaceous Wastes Processing and Process Intensification Research of Zhejiang Province, University of Nottingham Ningbo China, Ningbo, Zhejiang, 315100, China

8. Hefei National Laboratory for Physical Sciences at Microscale, CAS Key Laboratory of Materials for Energy Conversion, Department of Materials Science and Engineering, School of Chemistry and Materials Science, University of Science and Technology of China, Hefei, Anhui, 230026, China

9. These authors contributed equally.

*Corresponding Authors

Emails:

dewei.zhao@scu.edu.cn, xiangchaoyu@nimte.ac.cn, yong.ren@nottingham.edu.cn


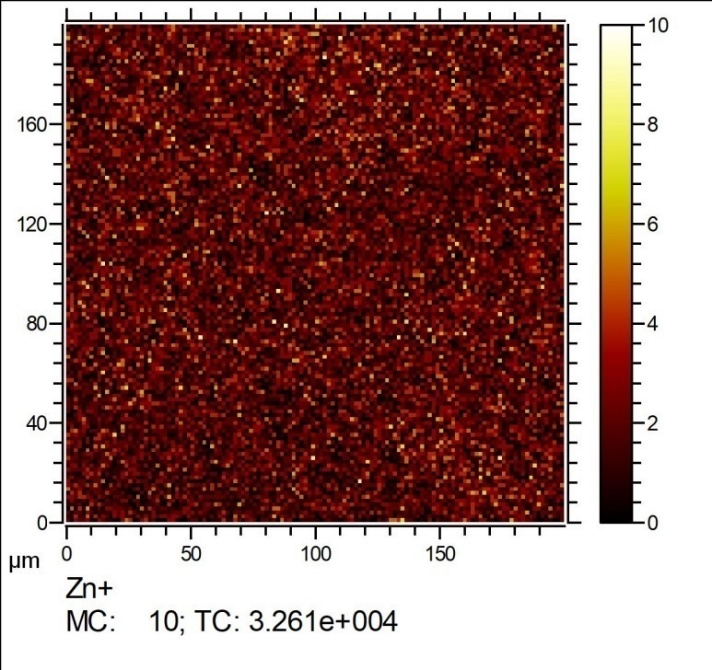


**Figure S1**. Zn^+^ element distribution characterization of the pristine:ZnBr_2_ perovskite film deposited on NiO/PVK.


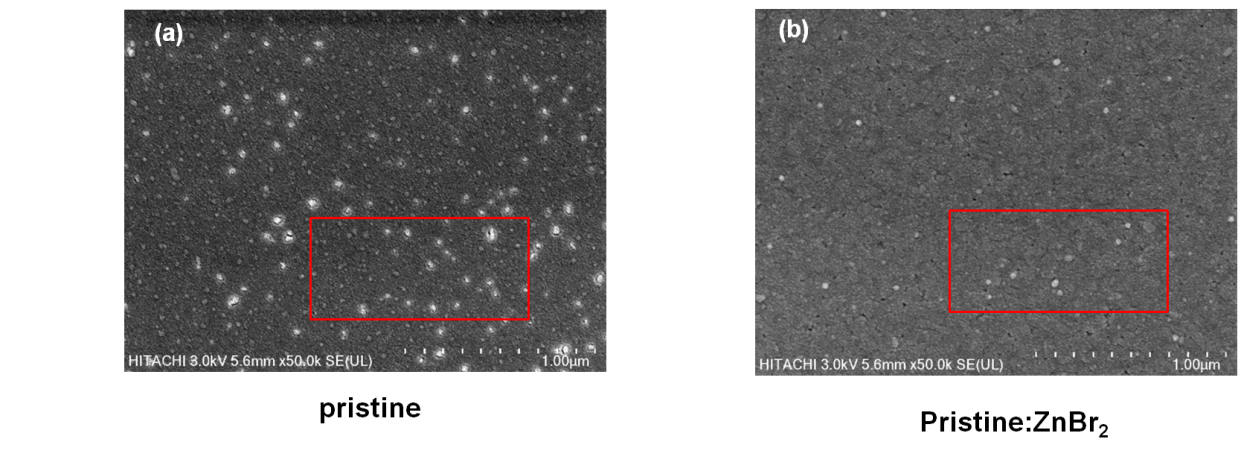


**Figure S2**. SEM images of the perovskite films deposited on ITO/NiO/PVK from two different precursor solutions: (a) pristine and (b) pristine:ZnBr_2_.


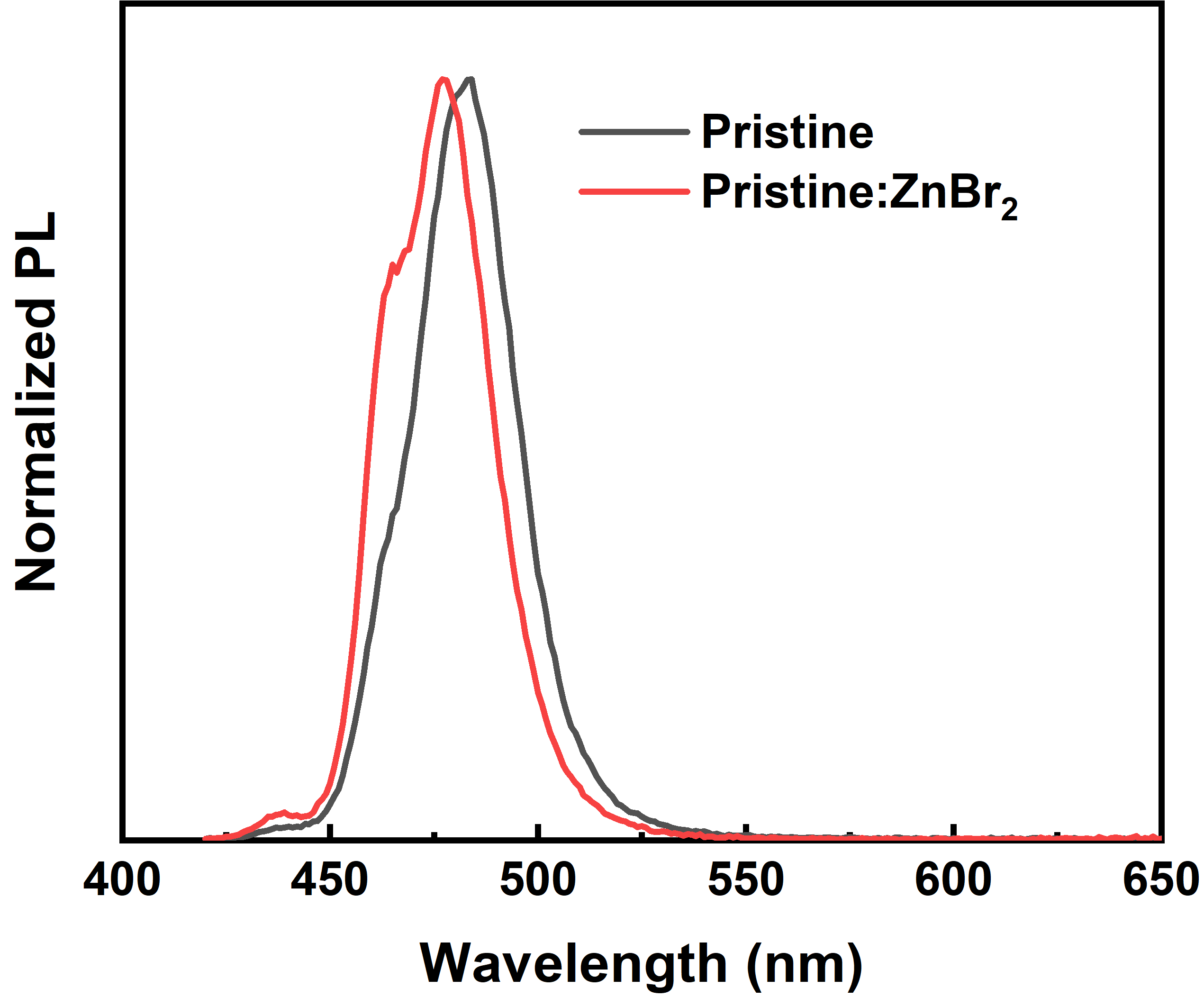


**Figure S3**. Steady-state PL of the pristine and pristine:ZnBr_2_ perovskite films coated on ITO/NiO/PVK.


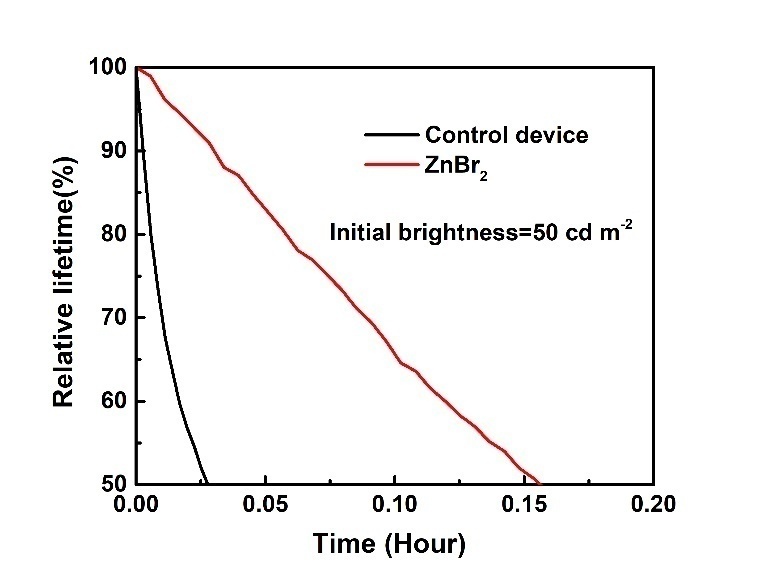


**Figure S4**. The T_50_ lifetimes of pristine and pristine:ZnBr_2_ PeLEDs under an initial brightness of ~50 cd m^-2^.

**Figure S5**. In-situ absorption measurement of quasi-2D perovskite formation without and with ZnBr_2_ during spin-coating ((a)-(b)) and intensity change at different wavelengths for different phases ((c)-(d)).


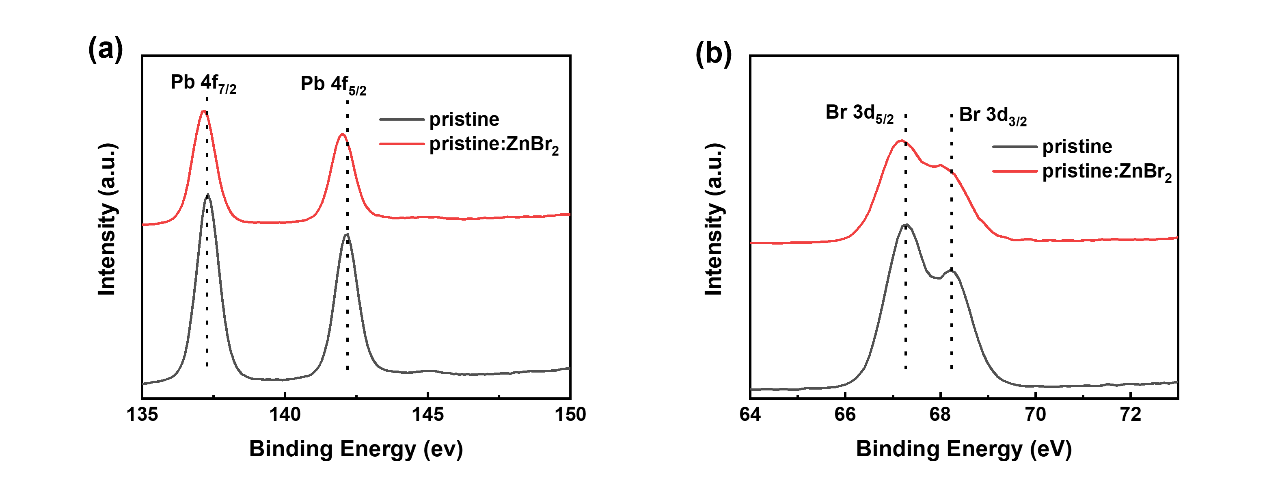


**Figure S6.** XPS spectra of Pb 4f and Br 3d signal for pristine and pristine:ZnBr_2_ perovskites.


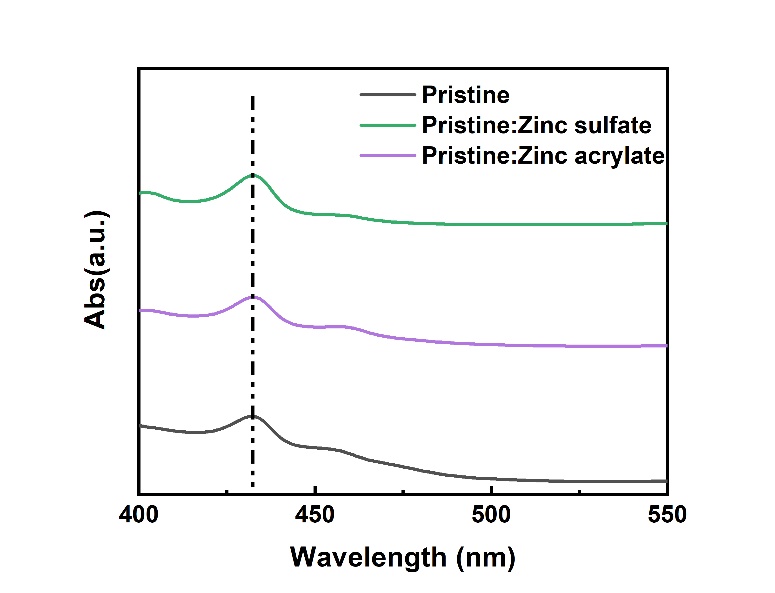


**Figure S7.** Steady-state absorption spectra for pristine, pristine: Zinc sulfate, pristine: Zinc acrylate based quasi-2D perovskite film.


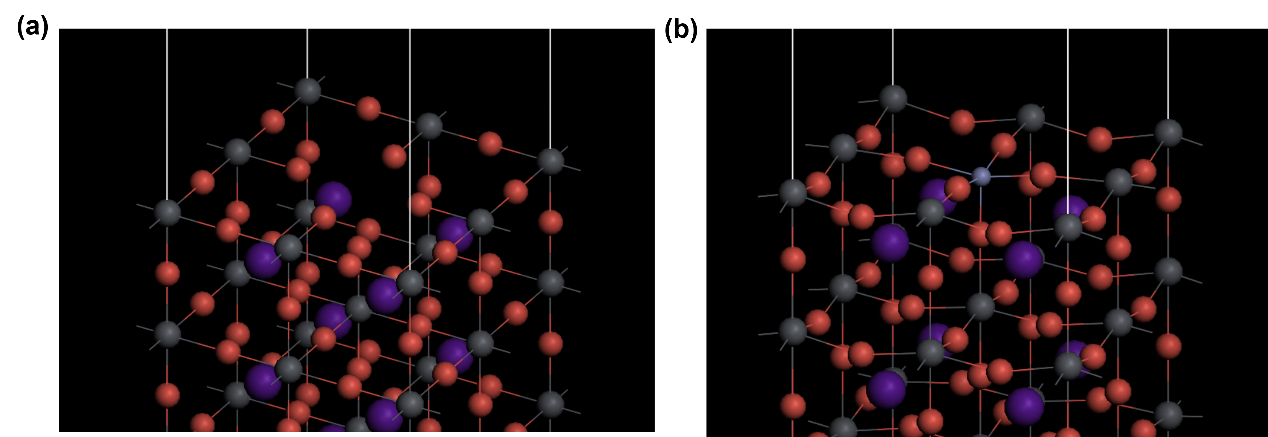


**Figure S8.** (a) Surface model of Pb vacancy. (b) Surface model of Zn passivated Pb vacancy (Grey=Pb Purple=Cs Brown=Br Grey-blue=Zn).


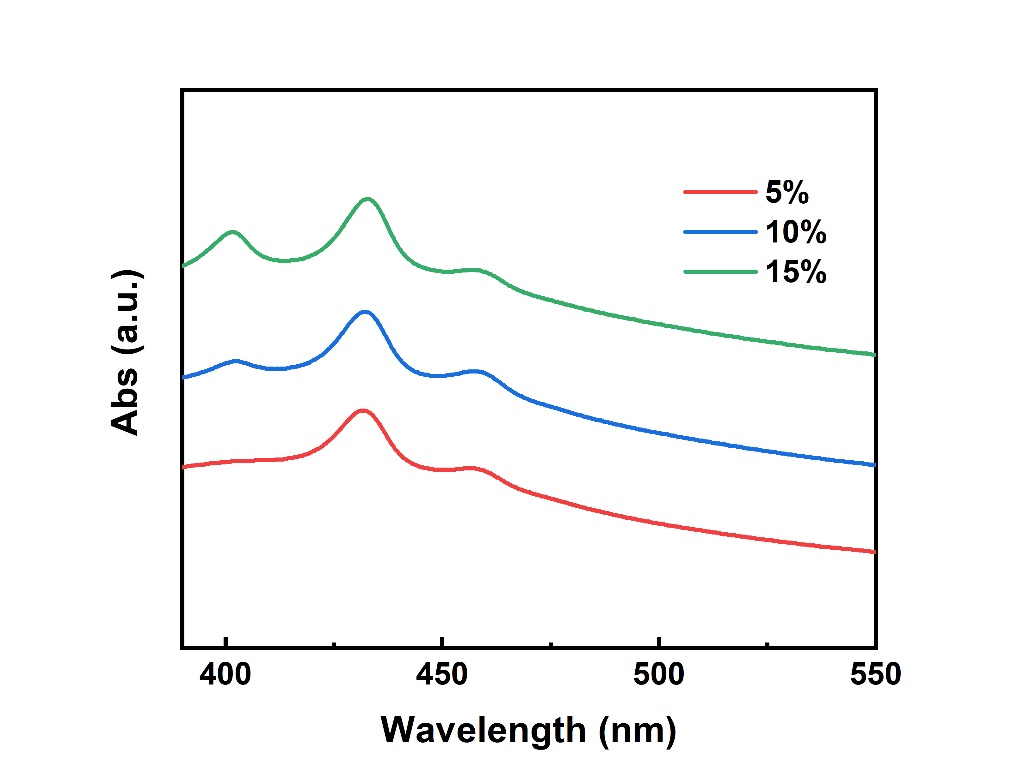


**Figure S9.** Absorption spectrum of quasi-2D perovskite films with different ZnBr_2_ (5%, 10%, 15%) doping amounts.


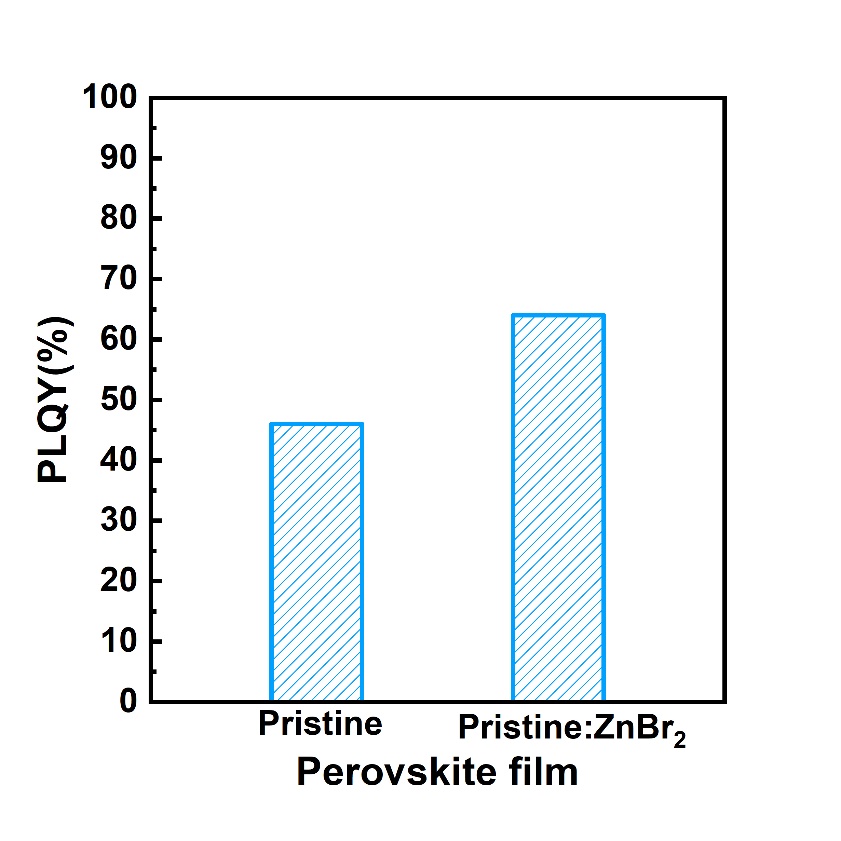


**Figure S10**. PLQY of the perovskite film coated on ITO/NiOx/PVK substrate (pristine and pristine:ZnBr_2_ sample).

**Table S1** TRPL fitting parameters of perovskite film without and with ZnBr_2_

| Perovskite film | τ_1_ (ns) | τ_2_ (ns) | τave (ns) |
| --- | --- | --- | --- |
| Pristine | 15.49 | 95.32 | 27.47 |
| Pristine:ZnBr_2_ | 20.24 | 127.9 | 41.77 |

**Table S2** Summary on the representative quasi-2D PeLEDs in blue region (480-495 nm) in literature.

| **Perovskite materials** | **EL (nm)** | **EQE (%)** | **Year** | **Ref.** |
| --- | --- | --- | --- | --- |
| PBABr_2_(Cs_0.7_FA_0.3_PbBr_3_) | 483 | 9.5 | 2019 | [1] |
| PEA:CsPb(Br_0.5_Cl_0.5_)_3_ | 485 | 11 | 2019 | [2] |
| PEA_2_(Cs_1-x_EA_x_PbBr_2_)_2_PbBr_4_ | 488 | 12.1 | 2020 | [3] |
| 5%YCl_3_-PEACl:CsPbBr_3_ | 488 | 13.5 | 2021 | [4] |
| PEA_x_PA_2−x_(CsPbBr_3_)_n−1_PbBr_4_:ABA | 486 | 10.1 | 2021 | [5] |
| PEA_x_PA_2−x_(CsPbBr_3_)_n−1_PbBr_4_:TBPO | 486 | 11.5 | 2022 | [6] |
| CsPb_1−x_Sr_x_Br_3_matrix:CsPbBr_3_ QD | 495 | 13.8 | 2022 | [7] |
| p-F-PEA:CsPb(Br_x_Cl_1-x_)_3_:PHDI | 487 | 14.8 | 2022 | [8] |
| GABA:PEABr:Cs_x_EA_1-x_PbBr_3_ | 490 | 15.6 | 2022 | [9] |
| DFBP:PEABr:CsPbBr_3_ | 490 | 15.03 | 2023 | [10] |
| BAP: p-F-PEABr:CsPb(Cl/Br)_3_ | 483 | 18.65 | 2023 | [11] |
| CsCl-PEA_2_(Cs_x_EA_1–x_PbBr_3_)_2_PbBr_4_ | 486 | 16.07 | 2023 | [12] |
| PABr:PEA_2_Cs_1.5_Pb_2.5_Br_8.5_ :ZnBr_2_ | **486** | **16.2** | **Our work** |  |

**References**

[1] Y. Liu, J. Cui, K. Du, H. Tian, Z. He, Q. Zhou, Z. Yang, Y. Deng, D. Chen, X. Zuo, Y. Ren, L. Wang, H. Zhu, B. Zhao, D. Di, J. Wang, R. H. Friend, Y. Jin, *Nat Photonics* **2019**, *13*, 760.

[2] Q. Wang, X. Wang, Z. Yang, N. Zhou, Y. Deng, J. Zhao, X. Xiao, P. Rudd, A. Moran, Y. Yan, J. Huang, *Nat Commun* **2019**, *10*, 5633.

[3] Z. Chu, Y. Zhao, F. Ma, C.-X. Zhang, H. Deng, F. Gao, Q. Ye, J. Meng, Z. Yin, X. Zhang, J. You, *Nat Commun* **2020**, *11*, 4165.

[4] Y. Liu, L. Zhang, S. Chen, C. Liu, Y. Li, J. Wu, D. Wang, Z. Jiang, Y. Li, Y. Li, X. Wang, B. Xu, *Small* **2021**, *17*, 2101477.

[5] Z. Ren, J. Yu, Z. Qin, J. Wang, J. Sun, C. C. S. Chan, S. Ding, K. Wang, R. Chen, K. S. Wong, X. Lu, W. Yin, W. C. H. Choy, *Advanced Materials* **2021**, *33*, 2005570.

[6] Z. Ren, J. Sun, J. Yu, X. Xiao, Z. Wang, R. Zhang, K. Wang, R. Chen, Y. Chen, W. C. H. Choy, *Nanomicro Lett* **2022**, *14*, 66.

[7] Y. Liu, Z. Li, J. Xu, Y. Dong, B. Chen, S. M. Park, D. Ma, S. Lee, J. E. Huang, S. Teale, O. Voznyy, E. H. Sargent, *J Am Chem Soc* **2022**, *144*, 4009.

[8] Y. Shen, Y. Li, K. Zhang, L. Zhang, F. Xie, L. Chen, X. Cai, Y. Lu, H. Ren, X. Gao, H. Xie, H. Mao, S. Kera, J. Tang, *Adv Funct Mater* **2022**, *32*, 2206574.

[9] S. Liu, Z. Guo, X. Wu, X. Liu, Z. Huang, L. Li, J. Zhang, H. Zhou, L. Sun, C. Yan, *Advanced Materials* **2023**, *35*, 2208078.

[10] B. Wang, Y. Zhou, S. Yuan, Y. Lou, K. Wang, Y. Xia, C. Chen, J. Chen, Y. Shi, Z. Wang, L. Liao, *Angewandte Chemie International Edition* **2023**, *62*, DOI 10.1002/anie.202219255.

[11] W. Zhou, Y. Shen, L. Cao, Y. Lu, Y. Tang, K. Zhang, H. Ren, F. Xie, Y. Li, J. Tang, *Adv Funct Mater* **2023**, *33*, DOI 10.1002/adfm.202301425.

[12] Z. Chu, W. Zhang, J. Jiang, Z. Qu, F. Ma, Y. Zhao, X. Chu, Y. Shen, Y. Li, Z. Yin, X. Zhang, J. You, *Nat Electron* **2023**, *6*, 360.
